# Differential vascular dysfunction in response to diets of differing macronutrient composition: a phenomenonological study

**DOI:** 10.1186/1743-7075-4-15

**Published:** 2007-06-14

**Authors:** Sameer Fatani, Lucy C Pickavance, Claire J Sadler, Joanne A Harrold, Roslyn Cassidy, John PH Wilding, Ebrahim K Naderali

**Affiliations:** 1 School of Clinical Sciences, University of Liverpool, Liverpool, UK; 2 Department of Veterinary Preclinical Sciences, University of Liverpool, Liverpool, UK; 3 School of Psychology, University of Liverpool, Liverpool, UK

## Abstract

**Background:**

Vascular dysfunction can develop from consumption of an energy-rich diet, even prior to the onset of obesity. However, the roles played by different dietary components remain uncertain. While attempting to develop models of obesity in a separate study, we observed that two high-energy diets of differing macronutrient compositions affected vascular function differently in overweight rats.

**Methods:**

Male Wistar rats (*n *= 6/group) were fed diets providing varying percentages of energy from fat and carbohydrate (CHO). For 10 weeks, they were fed either chow, as control diet (10% of energy from fat; 63% from CHO), chow supplemented with chocolate biscuit (30% fat; 56% CHO) or a high-fat diet (45% fat; 35% CHO). Blood concentrations of biochemical markers of obesity were measured, and epididymal fat pads weighed as a measure of adiposity. Mesenteric arteries were dissected and their contractile and relaxant properties analysed myographically. Data were tested by analysis of variance (ANOVA).

**Results:**

Weight gain and plasma concentrations of glucose, insulin and leptin were similar in all groups. However, biscuit-fed animals showed increased food intake (+27%; *p *< 0.01) and elevated concentrations of TGs and NEFAs (+41% and +17%; both *p *< 0.05). High-fat-fed animals showed an increase only in NEFAs (+38%; *p *< 0.01). Arterial vasoconstriction in response to NA and KCl increased only in biscuit-fed rats (both *p *< 0.01), while vasorelaxation in response to CCh and SNP, but not histamine, was attenuated in both groups (both *p *< 0.01). Furthermore, whereas the effect of the high-fat diet was most pronounced in endothelium-dependent vasorelaxation, the biscuit diet had the greater effect on endothelium-independent vasorelaxation.

**Conclusion:**

Vascular dysfunction resulting from consumption of a high-fat or combined relatively high-fat/high-CHO diet occurs through different physiological processes, which may be attributable to their differing macronutrient compositions. Combining potentially atherogenic macronutrients induces more extensive vascular impairment than that of high-fat alone, and may be attributable to the more marked dyslipidaemia observed with such a diet. Thus, these findings help clarify the role of dietary components in vascular impairment, which has implications for clinical approaches to preventing cardiovascular disease.

## Background

The link between obesity and vascular dysfunction is well-established [1-8], but its causes remain uncertain. Of the circulating factors increased in obesity – including leptin, insulin, NEFAs and TGs [5,6,9-14] – only the latter two are raised by short-term high-energy feeding (prior to obesity onset), suggesting they may play the more critical role [15-18]. Dissecting out the relative importance of dietary macronutrients in atherogenic dyslipidaemia is complex [19], but it is believed that carbohydrate plays the primary role by stimulating insulin secretion, downstream of which fatty acid metabolism is determined [20,21]. The mechanisms by which these dietary constituents' effects alter vascular function, particularly in animal models, is less clear, although some attempts have been made by different methods in humans [16,22]. In view of this, we took the opportunity to investigate these mechanisms during an exploratory study in which we compared a number of high-energy diets for their abilities to induce weight gain in rats.

## Methods

Male Wistar rats (192 ± 4 g) were fed diets of differing compositions for 10 weeks (*n *= 6/group). Controls received standard chow (CRM Biosure, Cambridge, UK; 'chow'group). One experimental group received chow supplemented with chocolate biscuit (McVitie's 'HobNobs', Ashby de la Zouch, Leics, UK; 'biscuit' group), and the other received a fat-enriched diet (Research Diets, Inc, New Brunswick, NJ, USA; 'high-fat' group; Table 1). Food intake was measured daily and body weight weekly.

**Table 1:** Dietary breakdown of macronutrients (% of energy)

**Diet**	**Total CHO**	**Complex CHO**	**Simple CHO**	**Total Fat**	**Saturated fat**	**MUFA**	**PUFA**	**Total protein**
**Chow**	63.0	57.7	5.3	10.3	1.7	3.0	5.6	28.1
**'Biscuit'**	55.9	37.2	18.7	30.0	12.9	10.6	5.4	15.0
**High-fat**	35.0	16.9	16.9	45.0	39.1	0.0	5.5	20.0

At termination, fasting blood samples were collected and plasma glucose, NEFA and TG concentrations determined by diagnostic kit, and insulin and leptin by RIA. A single epididymal fat pad was dissected from each animal and weighed as a measure of adiposity. Length-tension characteristics of mesenteric arteries (6/rat) were determined, followed by evaluation of arterial contractility and relaxation, as described previously [18].

Before statistical analysis, vascular reactivity data were quantified as AUC. Experimental groups were compared on all parameters to controls by one-way ANOVA followed by *post hoc *analysis.

## Results

Biscuit-fed rats showed a higher daily energy intake and increased TG concentrations. The high-fat diet increased NEFAs by twice the proportion of the biscuit diet. There were non-significant rises in body weight and epididymal fat mass on both experimental diets, but neither affected glucose, insulin or leptin concentrations (Table 2).

**Table 2:** Body weight, energy intake and metabolic parameters

	**Chow**	**Biscuit**	**High-fat**
**Daily energy intake (kJ/d)**	361 ± 15	459 ± 14**	390 ± 13
**Body weight gain (g)**	299 ± 22	347 ± 15	339 ± 22
**Epididymal fat mass (g)**	4.5 ± 0.6	7.7 ± 0.8	6.6 ± 2.2
**Glucose (mM)**	9.8 ± 1.0	8.9 ± 1.7	9.3 ± 1.0
**Insulin (μU/ml)**	22.2 ± 2.6	21.1 ± 1.2	19.4 ± 1.6
**Leptin (ng/ml)**	5.3 ± 0.9	6.3 ± 0.3	5.9 ± 0.4
**Triglycerides (mg/dL)**	89.4 ± 4.8	126.5 ± 10.1*	77.7 ± 3.6
**NEFAs (mM)**	0.29 ± 0.01	0.34 ± 0.01*	0.40 ± 0.02**

*p < 0.05; **p < 0.01 *vs *chow

**Figure 1:**
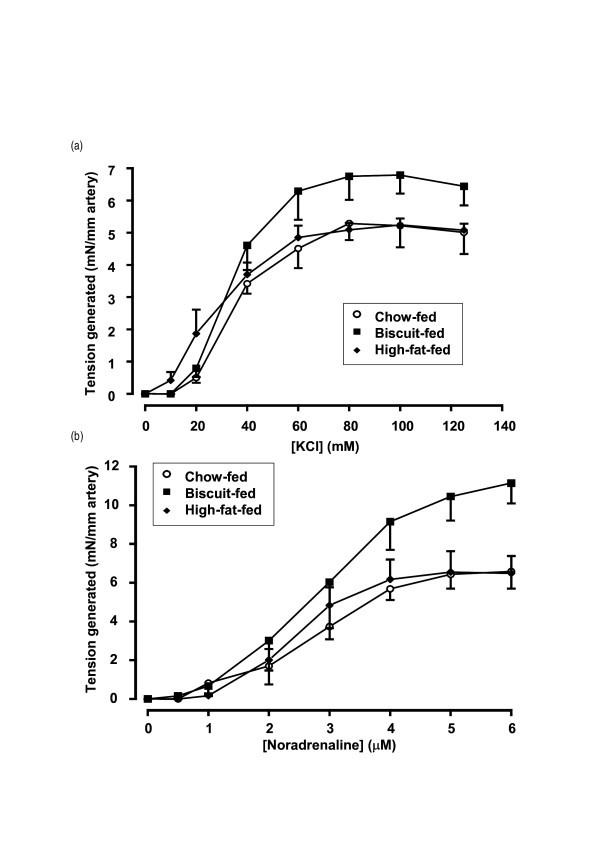
Arterial contractility.

The experimental diets did not alter vessel diameter (data not shown; both *p *> 0.05 *vs *chow). Both KCl and NA, however, increased vasoconstriction in the biscuit group (*p *< 0.01 and *p *< 0.001), but had no effect in the high-fat group (both *p *> 0.05; Fig. 1).

**Figure 2:**
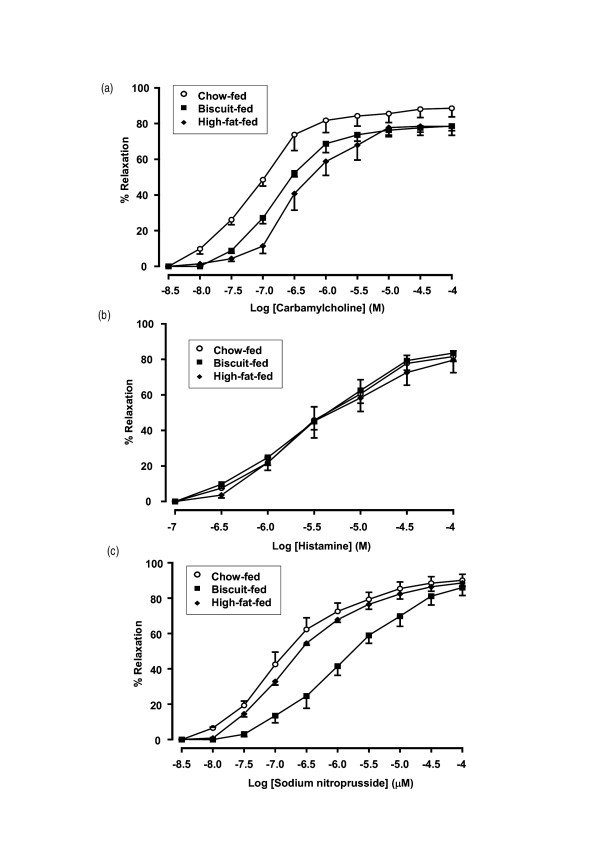
Arterial relaxation.

In response to cumulative addition of CCh (10 nM-100 μM), NA-pre-constricted arteries from biscuit- and high-fat-fed rats displayed a significant rightward shift of concentration-response curves, compared with controls (EC_50 _biscuit: 0.28 ± 0.01; high-fat: 0.52 ± 0.04 μM; both *p *< 0.001 *vs *chow: 0.11 ± 0.01 μM; Fig. 2a). Maximal vasorelaxation to 100 μM CCh was reduced by 13% in both biscuit- and high-fat-fed rats (both *p *< 0.01 *vs *chow-fed), but was more attenuated in the latter at lower CCh concentrations (3.16–316 nM; *p *< 0.01 *vs *biscuit; Fig. 2a). Histamine-induced vasorelaxation was similar in all groups (Fig. 2b).

The endothelium-independent vasodilator, SNP, also induced concentration-dependent vasorelaxation in arteries from all groups, the curves shifted to the right in the biscuit- and high-fat-fed groups (EC_50 _biscuit: 1.58 ± 0.02; high-fat: 0.24 ± 0.01; both *p *≤ 0.01 *vs *chow: 0.13 ± 0.02). However, maximum SNP-induced responses were similar in all groups. Compared with the chow- and high-fat-fed groups, SNP-induced relaxation in biscuit-fed rats was attenuated by more than 2-fold (both *p *< 0.001). Attenuation of SNP responses was also significant, though less pronounced, in high-fat-fed animals (*vs *control: *p *< 0.05; Fig. 2c).

## Discussion

This study confirms previous observations that high-energy diets lead to vascular dysfunction [5,6,17,18,23,24]. The high-fat and biscuit diets induced similar degrees of modest weight gain and adiposity despite different macronutrient compositions, suggesting these account for the differences observed in vascular reactivity. The biscuit diet had a detrimental effect on both vasoconstriction, and -relaxation, whereas the high-fat diet affected only the latter. The attenuation of vasorelaxation in response to the cGMP-mediated actions of both CCh and SNP shows that this effect of both diets is mediated through both endothelium and vascular smooth muscle. Consumption of other high-energy diets has shown this dual process [5,17,18], although some such diets exert their effects only through one mechanism [23], further highlighting the specificity of effects depending on dietary components. The failure of either diet to alter cAMP-mediated vasorelaxation (i.e., histamine-induced responses) also appears to be a common feature of high-energy diets [17,18].

Intriguingly, the degree of abnormality seen in both endothelium- and smooth muscle-mediated vasodilation also differed according to diet. CCh-induced vasorelaxation was more severely attenuated by the high-fat diet, and the SNP response more so by the biscuit diet. These findings suggest that different dietary components affect vasorelaxation by different processes, that a diet high in fat has greater deleterious effects on the arterial endothelial lining, whereas a diet relatively rich in carbohydrate *and *fat largely compromises the vascular smooth muscle. There is substantial supporting evidence that dietary components play a role in vascular function. Humans and animals fed diets high in fat, particularly saturated fat [19,24-26], show endothelial abnormalities. On the other hand, reports of the protective effects of high-carbohydrate (low-saturated fat) diets and diets high in unsaturated fats are contentious [16,19,22,27,28]. The biscuit diet, although lower in both total and saturated fat than the high-fat diet, contained a significantly greater amount of MUFAs. Although these are associated with improved cardiovascular risk [27,29], at high levels they can acutely impair endothelial function [16]. Recent reviews of the literature note that stimulation of insulin release by increased carbohydrates promotes adipogenesis, weight gain and atherogenesis, all associated with the metabolic syndrome [19,28,30]. The biscuit diet was higher in overall carbohydrate content than the high-fat diet, and it may be this, in combination with relatively high fat content, that accounts for its more detrimental effects. The relatively lower saturated fat and higher carbohydrate content may also be more palatable and account for the increased daily intake in the biscuit-fed animals, and, hence, epididymal adiposity (though short of significance) and overall greater hyperlipidaemia. This would have to be more carefully analysed in future, however, by matching intake volume and overall energy content between groups. An adipocentric view would suggest that the increased adiposity *per se *of biscuit-fed animals is critically linked with the worsened vascular defects in this group, with excessive fat mass in this depot resulting in fat cell dysfunction, which in turn contributes to the metabolic disorders that increase the risk of atherosclerosis [31].

High-carbohydrate meals may also stimulate sympathetic nervous system activity *in vivo *[32], and counteract insulin-induced vasodilation. Although fasting insulin levels were similar in all groups, it is possible that differences in post-prandial insulin (which would be expected to be higher in rats fed a carbohydrate-enriched diet and resulting adipogenesis) and then raised plasma TGs, resulting in part from *de novo *lipogenesis, may account for the vascular differences. Indeed, hypertriglyceridaemia is a recognized atherogenic risk factor (e.g., [5,19,33]). This can be the case even in the absence of insulin resistance [5] or symptoms of atherosclerosis [34]. Although our animal model did not adequately mimic human obesity in terms of weight gain and some of its metabolic disturbances – probably due in part to a lack of statistical power – it remains predictive of vascular dysfunction in the absence of these and therefore further highlights the insidiousness of the disorder.

Finally, although % energy derived from protein was lower in both biscuit and high-fat diets compared to chow, the total average daily protein intake was similar (67 g and 78 g/day, respectively; biscuit *vs *high-fat: *p *> 0.05). Hence, it is unlikely that protein deficiency explains the differences in vascular function. Dietary antioxidant levels may also play a role (e.g., [35,36]), but it is not possible to comment further on these as they were not measured.

## Conclusion

The limitations of this study, therefore, revolve around the fact that the nutrient contents of the diets were not sufficiently controlled to draw very precise conclusions regarding their comparative effects. We cannot know whether their differential effects on vascular function were direct effects of nutrient content or indirect effects of resulting adiposity and/or circulating lipids. However, this study does show that high-energy diets of varying compositions can induce vascular dysfunction to varying degrees in the rat via mechanisms involving different layers of the vascular wall. The combination of high-fat and high-carbohydrate diets may be particularly damaging, possibly through increased hyperlipidaemia.

## Abbreviations

ANOVA: analysis of variance

AUC: area under the curve

CCh: carbamylcholine

CHO: carbohydrate

KCl: potassium chloride

MUFA: monounsaturated fatty acid

NA: noradrenaline

NEFA: non-esterified fatty acid

PUFA: polyunsaturated fatty acid

RIA: radioimmunoassay

TG: triglyceride

SNP: sodium nitroprusside

## Competing interests

The author(s) declare that they have no competing interests.

## Authors' contributions

SF carried out the myography and participated in tissue collection, statistical analysis and drafting of the manuscript.

LCP calculated the diet composition and participated in data collection, tissue sampling, statistical analysis and drafting of the manuscript.

CJS participated in data collection, tissue sampling and statistical analysis, and carried out metabolic assays.

JAH participated in data collection, tissue sampling and statistical analysis, and carried out metabolic assays.

RC participated in data collection and tissue sampling.

JPHW participated in drafting of the manuscript.

EKNA participated in the conception and design of the vascular study, the myography and in drafting of the manuscript.

All authors read and approved the final manuscript.
